# Fecal microbiota transplantation restores normal fecal composition and delays malignant development of mild chronic kidney disease in rats

**DOI:** 10.3389/fmicb.2022.1037257

**Published:** 2022-11-30

**Authors:** Xiaoxue Liu, Ming Zhang, Xifan Wang, Ping Liu, Longjiao Wang, Yixuan Li, Xiaoyu Wang, Fazheng Ren

**Affiliations:** ^1^Key Laboratory of Functional Dairy, Co-Constructed by Ministry of Education and Beijing Municipality, College of Food Science and Nutritional Engineering, China Agricultural University, Beijing, China; ^2^School of Food and Chemical Engineering, Beijing Technology and Business University, Beijing, China; ^3^Key Laboratory of Precision Nutrition and Food Quality, Department of Nutrition and Health, China Agricultural University, Beijing, China; ^4^Food Laboratory of Zhongyuan, Luohe, Henan, China

**Keywords:** fecal microbiota transplantation, chronic kidney disease, protein-bound uremic toxins, lysine production, metagenomics

## Abstract

Chronic kidney disease (CKD) is associated with gut microbiome dysbiosis, but the role of intestinal flora in CKD treatment remains to be elucidated. Fecal microbiota transplantation (FMT) can be utilized to re-establish healthy gut microbiota for a variety of diseases, which offers new insight for treating CKD. First, 5/6 nephrectomy rats (Donor CKD) and sham rats (Donor Sham) were used as donors for FMT, and fecal metagenome were analyzed to explore potential therapeutic targets. Then, to assess the effect of FMT on CKD, sterilized 1/2 nephrectomy rats were transplanted with fecal microbiota from Donor sham (CKD/Sham) or Donor CKD (CKD/CKD) rats, and 1/2 nephrectomy rats without FMT (CKD) or no nephrectomy (Sham) were used as model control or normal control. Results showed that *Bacteroides uniformis* and *Anaerotruncus* sp. *1XD22-93* were enriched in Donor CKD, while *Lactobacillus johnsonii* and *Lactobacillus intestinalis* were reduced. In addition, the increased abundance of microbial functions included tryptophan metabolism and lysine degradation contributing to the accumulation of protein-bound uremic toxins (PBUTs) in Donor CKD. Genome analysis indicated that FMT successfully differentiated groups of gut microbes and altered specific gut microbiota after 1 week of treatment, with *Bacteroides uniformis* and *Anaerotruncus* sp. *1XD22-93* increasing in CKD/CKD group as well as *Lactobacillus johnsonii* and *Lactobacillus intestinalis* being improved in CKD/Sham group. In comparison to CKD group, substantial PBUT buildup and renal damage were observed in CKD/CKD. Interestingly, compared to CKD or CKD/CKD group, tryptophan metabolism and lysine degradation were efficiently suppressed in CKD/Sham group, while lysine biosynthesis was promoted. Therefore, FMT considerably reduced PBUTs accumulation. After FMT, PBUTs and renal function in CKD/Sham rats remained the same as in Sham group throughout the experimental period. In summary, FMT could delay the malignant development of CKD by modifying microbial amino acid metabolism through altering the microenvironment of intestinal flora, thereby providing a novel potential approach for treating CKD.

## Introduction

Chronic kidney disease (CKD) is estimated to affect one sixth of the population worldwide, which causes significant morbidity, mortality, and severe socio-economic burden (Goolsby, 2002; Jha et al., 2013; Bikbov et al., 2020). The damaged kidney is unable to filter toxic terminal metabolites, resulting in increased circulating toxin (Chung et al., 2019). These toxic retention solutes were associated with worsening outcomes in CKD patients, in particular with cardiovascular morbidity and mortality (Meyer and Hostetter, 2007; Al Khodor and Shatat, 2017; Mahmoodpoor et al., 2017). Especially, protein-bound uremic toxin (PBUT) is the most destructive toxin and the most difficult to be removed clinically in CKD development (Itoh et al., 2012; Sirich and Meyer, 2018). PBUTs are mainly produced from the metabolism of aromatic amino acids by gut bacteria (Wikoff et al., 2009; Gryp et al., 2020). Such as indoxyl sulfate (IS), the most well-studied PBUT, is produced by gut microbials metabolizing tryptophan (Mishima et al., 2017). Interventions for intestinal flora have been widely studied to decrease PBUTs (Chen et al., 2022). Hence, microbiota-driven therapy aimed at decreasing the circulating PBUT concentration which could provide a promising new therapeutic strategy for patients with CKD.

In recent decades, a great deal of effort has focused on the differences in gut microbial composition between CKD patients and healthy controls, confirming the relationship between gut bacterial and PBUTs (Vaziri et al., 2013; Wang et al., 2020). To alleviate the progression of CKD, several therapies targeting gut flora have been reported. AST-120, an oral charcoal adsorbent, is a classic solution for the continuous reduction of some PBUTs in hemodialysis patients (Yamamoto et al., 2015). However, the poor acceptability of AST-120 with long-term administration may affect essential nutrient absorption due to non-specific capture. In the meantime, the modulation of probiotic supplement is also an attractive strategy to inhibit PBUTs formation (e.g., IS and p-cresyl sulfate) in CKD patients (Guida et al., 2014; Rossi et al., 2016). However, previous studies have reported that probiotics treatment only partially reduced some kind of PBUTs. In addition, the ideal mix of bacterial strains, dosage, and administration time for the reduction of PBUTs still remain unknown. Therefore, it is still unclear how to reduce total PBUTs by regulating gut microbiome dysbiosis in a healthy and sustained manner.

Fecal microbiota transplantation (FMT), a way to reconstitute receptor gut microbiota by transplanting of stool from a volunteer (D’Haens and Jobin, 2019), offers a potential therapeutic approach for CKD. Multiple studies have shown that transplanting healthy gut microbiota plays a therapeutic role in other kidney diseases. A clinical case study reported that FMT could cure the nephritis caused by gut microbiota producing extended spectrum beta-lactamase (Singh et al., 2014). In studies investigating immunoglobulin A (IgA) nephropathy, it was reported that FMT decreased inflammation and 24 h urinary protein, and increased serum albumin (Lauriero et al., 2021; Zhao et al., 2021). Meanwhile, among the various published studies, FMT has been a helpful tool to verify the correlation between gut dysbiosis and CKD progression, which implies its value in restoring gut microbiota in CKD (Cai et al., 2020; Li et al., 2020; Han et al., 2021). However, little is known about the application of FMT in the treatment of CKD at present.

In this study, flora from healthy and severe CKD rats were transplanted into a rat model of antibiotic-treated mild CKD. By interaction analysis of fecal metagenomic and serum PBUTs metabolome, it was determined how gut microbiome affected the development of CKD by PBUTs and whether transplantation from healthy colonic bacteria delayed the onset of CKD progression.

## Materials and methods

### Preparation of donor-rats for fecal microbiota transplantation

Animal experiments were approved by the Animal Care and Use Committee of China Agricultural University. A total of 35 male CD^®^ (SD) IGS Rats (Beijing Vital River Laboratory Animal Technology Co., Ltd., Beijing, China) were given a standard diet and housed under standard conditions. Rats were acclimatized and fed for 7 days before performing the experiments described below.

**FIGURE 1:**
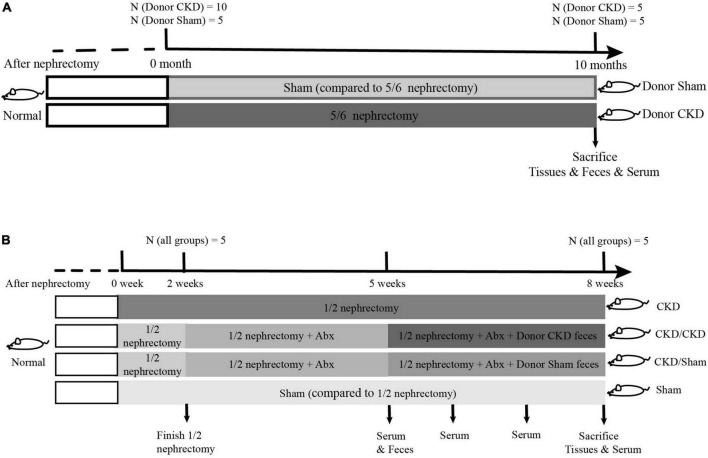
The graphical scheme of the animal experiment. **(A)** Preparation of donor-rats for fecal microbiota transplantation. **(B)** Experimental design of fecal microbiota transplantation.

To obtain feces using for FMT, fifteen rats at 6 weeks old were randomly divided into two groups for 5/6 nephrectomy or sham surgeries: a 5/6 nephrectomy group (Donor CKD, *n* = 10) and a sham-operated group (Donor Sham, *n* = 5). The 5/6 nephrectomy was a bilateral nephrectomy and was performed in two stages. Firstly, 2/3 of the left kidney was removed, and then the whole right kidney was removed. The sham-operated group was used as a control for the 5/6 nephrectomy group, which was performed twice as same as the 5/6 nephrectomy to exclude the interference of the laparotomy operation. After 10 months of CKD progression, half of the rats in the Donor CKD group died. The 5/6 nephrectomy rats developed severe CKD by this point. Thus, the remaining nephrectomy rats and sham rats were donors of gut microbiota for the following experiment (Figure 1A).

For FMT preparation, donor rats were placed in empty autoclaved cages (without bedding) and allowed to defecate freely. Donor feces were obtained simultaneously from all donor rats in each group, rather than from individual rat. Only feces that were not contaminated with pee were collected from the two groups of rats. Two sets of fecal samples from donors were placed on ice during the collection. After being returned to the laboratory, a final dilution with sterile saline (1:5, m: v) is performed in a sterile setting. Finally, fecal suspensions were mixed with 10% sterile glycerol and stored at –80°C until further analyzed.

### Experimental design of fecal microbiota transplantation

To determine whether FMT has beneficial effect on mild CKD, 26-week-old rats were randomly subjected to 1/2 nephrectomy (to remove one kidney, *n* = 15) or sham surgery (Sham group, only one operation, *n* = 5). After 1-week operation convalescent period, 10 rats in the 1/2 nephrectomy group were treated with a 3-week course of mixed antibiotics treatment (ampicillin, metronidazole, neomycin, and vancomycin according to the ratio 2:2:2:1) to deplete gut microbiota (Wang et al., 2011). During antibiotic treatment, the feces of antibiotic-treated rats were collected weekly for confirmation of intestinal sterility. After determining intestinal sterility, rats treated with antibiotics were randomly assigned into two FMT recipient groups. One group received intestinal flora from Donor Sham (CKD/Sham, *n* = 5) and the other received intestinal flora from Donor CKD (CKD/CKD, *n* = 5). FMT was performed by gavage daily (1 mL, once per day) for 3 weeks. The remaining 1/2 nephrectomy rats served as model control, while sham-operated rats served as healthy control for 1/2 nephrectomy rats (Figure 1B).

### Metabolic parameters

Body weight, serum level of creatinine (Crea), blood urea nitrogen (BUN), and PBUTs were measured. From the beginning to the end of FMT, serum renal function indicators were measured weekly. Each group of rats was fasted overnight prior to blood collection. Crea and BUN contents were evaluated using a BS-420 automated biochemical analyzer (Mindray, China). Six main PBUTs in serum such as indoxyl sulfate (IS), p-cresyl sulfate (PCS), phenyl sulfate (PS), hippuric acid, trimethylamine N-oxide (TMAO), and phenylacetyl glycine, were measured at the experimental end point by Bruker RP-HPLC with a Waters HSS T3 column (2.1 × 100 mm, 1.8 μm) (Wang et al., 2020). The quantitation of PBUTs was performed by constructing calibration curves using the standard PBUTs (TRC, Inc., CA, and San Diego, USA).

### Microbiota analyses

In this study, the gene abundance and functional pathways at the taxonomic level for every bacterial species were determined using metagenomic sequencing of all genes present in the gut microbiome. The raw data was analyzed by online platform. (Majorbio Biomedical Technologies Ltd., China) (Ren et al., 2022).

And then, the raw reads from metagenome sequencing were removed adaptor sequences, trimmed and quality-controlled to generate the clean reads using “fastp” (Chen et al., 2018) on the free online platform of Majorbio Cloud Platform. Using the de Bruijn graphs–based assembler MEGAHIT (Li et al., 2015), these high-quality reads were subsequently combined into contigs. As a final result, contigs with the length being or more than 300 bp were selected.

Representative sequences of the non-redundant gene catalog were annotated using the blastp program of DIAMOND v0.9.19 against the NCBI NR database with an *e*-value cutoff of 1e^–5^ (Buchfink et al., 2015). With the same *e*-value cutoff, the metabolic and functional pathways were annotated based on the KEGG databases by running Diamond against the Kyoto Encyclopedia of Genes and Genomes.

### Histological analyses

Part of the kidney was put in 4% paraformaldehyde at room temperature for 24 h (Servicebio, China) and paraffin-embedded. Hematoxylin & eosin (H&E), Masson, and PASM stains were done using staining kits (Servicebio, China) after 5-μm thick slices were cut. After staining, the tissue structure was observed under a light microscope and recorded.

### Western blot

Kidney protein was extracted using total protein extraction kit (BestBio, China). The concentration of protein extract was determined using standard BCA method (Beyotime, China). Proteins were loaded onto 5–8% gradient SDS-PAGE gels (CWBIO, China) for electrophoresis and transferred to PVDF membranes (0.45 μm, Millipore, US). And then the membranes were treated overnight at 4°C with the primary antibody against NADPH oxidase (ab133303, 1:2,000, Abcam, US). Next, membranes were washed and treated with secondary antibodies linked to horseradish peroxidase (A0208, 1:1,000, Beyotime Biotechnology, China). Subsequently, enhanced chemiluminescence compounds were used to create signals (EMD Millipore, US).

### Statistical analysis

Results were expressed as mean ± SEM, and data analysis was performed using SPSS 21.0 software. Differences between groups were analyzed by *t*-test and one-way ANOVA. Free online platform Majorbio Cloud^1^ was used for metagenome analysis. Microbiota data were analyzed and graphed by the non-parametric tests Kruskal-Wallis sum rank test and Wilcoxon rank sum test. *P* < 0.05 was considered statistically significant.

## Results

### Alteration in gut microbial composition was associated with chronic kidney disease

**FIGURE 2:**
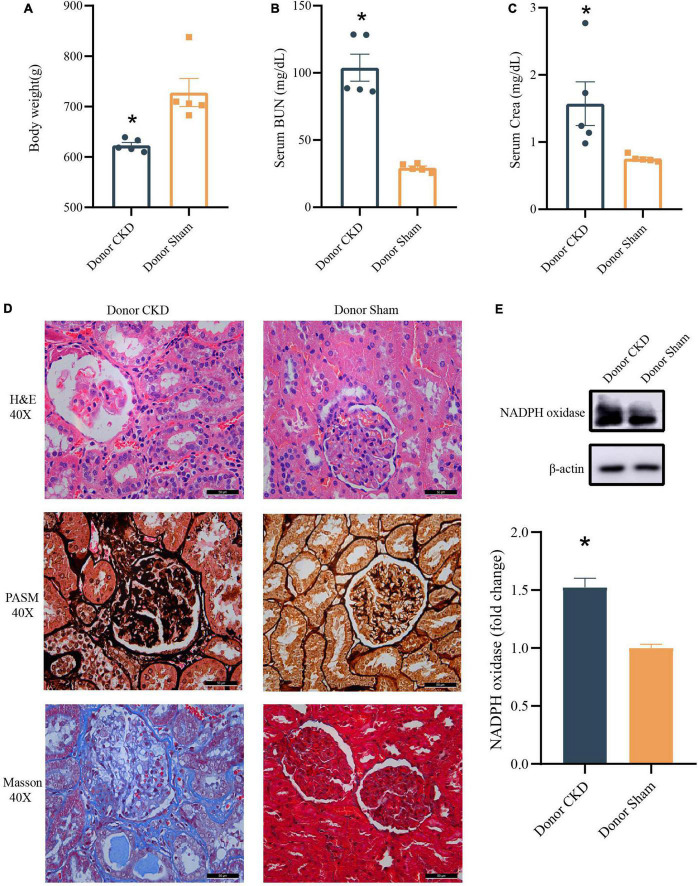
Changes in various indicators after successful establishment of a kidney disease model. **(A)** Body weight. **(B)** BUN. **(C)** Crea. **(D)** Histology of kidney tissue stained with H&E, PASM, and Masson. **(E)** Western blot results for NADPH oxidase. The data is presented as means ± SEM. Donor Sham, *n* = 5; Donor CKD, *n* = 5. *p < 0.05.

To confirm the establishment of the Donor CKD model, the physiological indicators associated with the nephropathy were observed. The Body weight of Donor CKD was lower than Sham group with significant difference (*P* < 0.05) (Figure 2A). Moreover, serum BUN and Crea levels were nearly twice as high in the Donor CKD group as in the Donor Sham group (*P* < 0.05) (Figures 2B,C). Pathological signs of renal damage and fibrosis were also evaluated. In the Donor CKD group, staining revealed tubular atrophy, glomerular sclerosis, interstitial infiltration, and overall renal fibrosis (Figure 2D). Finally, rats in CKD showed significant increased NADPH oxidase activity (*P* < 0.05), suggesting greater renal oxidative damage (Figure 2E). These observations indicate that CKD donor model was successfully established.

**FIGURE 3:**
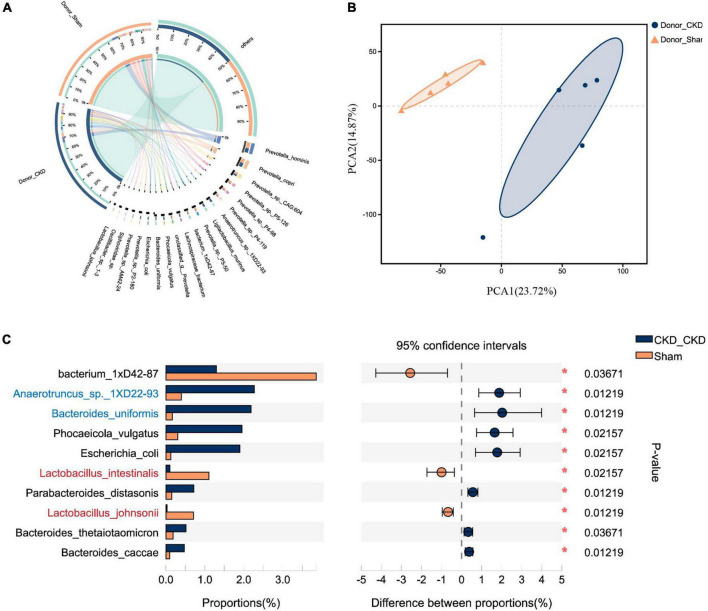
Differences in gut microbiota between Donor CKD and Donor Sham rats. **(A)** Circos sample-species relationship map. **(B)** PCA plots of species-level metagenomic expression (PERMANOVA *P* < 0.05). **(C)** Differential microbial species (Wilcoxon rank sum test). The vertical coordinates indicate the species names at different taxonomic levels, and the horizontal coordinates indicate the percentage value of the abundance of a species for that sample, with different colors indicating different groupings. Donor Sham, *n* = 5; Donor CKD, *n* = 5. *P < 0.05.

To corroborate the taxonomic features of the intestinal flora between CKD and normal rats, metagenomic analysis on donors was performed (Figure 3). A total of 16,152 species-related metagenomic findings were collected. A Circos plot clearly showed that differing amounts of the same species appearing between Donor CKD and Donor Sham (Figure 3A). Furthermore, the principal component analysis (PCA) plots showed that the colony flora of the Donor CKD and Donor Sham were separated suggesting differences in bacterial composition (PERMANOVA *P* < 0.05) (Figure 3B). Statistical analysis by differential microbiota revealed that species most different in Donor Sham included *Lactobacillus johnsonii* and *Lactobacillus intestinalis* (*P* < 0.05), while *Anaerotruncus* sp. *1XD22-93* and *Bacteroides uniformis* were most enriched in Donor CKD (*P* < 0.05) (Figure 3C). Taken together, rats with CKD had significantly different gut flora from healthy rats.

### Disturbance in chronic kidney disease intestinal flora led to changes in bacterial amino acid metabolism and accumulation of protein-bound uremic toxins

**FIGURE 4:**
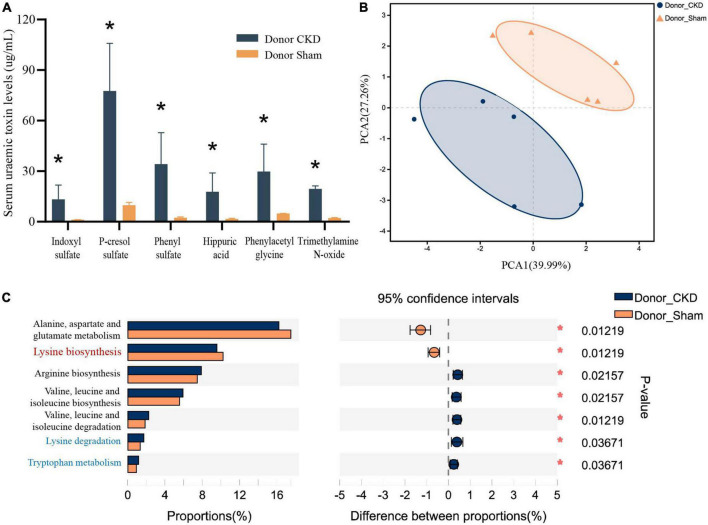
Differences in bacterial amino acid metabolism associated with the KEGG database between Donor CKD and Donor Sham rats. **(A)** PBUTs levels after successful establishment of a kidney disease model. **(B)** PCA plots of amino acid metabolism expression (PERMANOVA *P* < 0.05). **(C)** Differential amino acid metabolism pathways in gut microbiota (Wilcoxon rank sum test). The vertical coordinates indicate the KEGG function names at different classification levels, and the horizontal coordinates indicate the percentage value of a particular KEGG function abundance for that sample, with different colors indicating different groupings. Donor Sham, *n* = 5; Donor CKD, *n* = 5. *P < 0.05.

Major PBUTs were accumulated and showed a significant fold increase (*P* < 0.05) with renal failure in Donor CKD group (Figure 4A). A strong relationship was discovered between intestinal metagenomic species (MGSs) and serum PBUT levels (*r* = 0.45286, *P* = 0.008) through analysis of two donor groups (Table 1). The KEGG functional sequences in MGSs were matched to the KEGG database for amino acid metabolism since PBUT production is connected to colon bacteria amino acid metabolism (*r* = 0.42612, *P* = 0.022) (Table 1). Results showed that gut microbiota amino acid metabolism differed significantly between Donor CKD and Donor Sham (Figure 4B). Based on differential analysis, the tryptophan metabolism reported to be linked to PBUTs was dramatically elevated in the Donor CKD group (*P* < 0.05) (Figure 4C). The more surprising correlation was the significant variations in lysine-related metabolism between Donor CKD and Donor Sham group (Figure 4C). Lysine biosynthesis was significantly enriched in Donor Sham group (*P* < 0.05), whereas lysine degradation was higher in Donor CKD group (*P* < 0.05). These results suggested that lysine metabolism, similar to tryptophan metabolism, played an equally important role in CKD. Taken together, these data indicate that variations in PBUTs are closely related to changes in amino acid metabolism caused by differences in intestinal flora.

**TABLE 1:** Correlation of mantel test in donors.

dm1	dm2	Mantel r	*P*-value	Permutations	Tail type
Species matrix	Toxin matrix	0.45286	0.008	999	Two-sided
Amino acid metabolism matrix	Toxin matrix	0.42612	0.022	999	Two-sided

### Fecal microbiota transplantation restored normal fecal composition in chronic kidney disease

**FIGURE 5:**
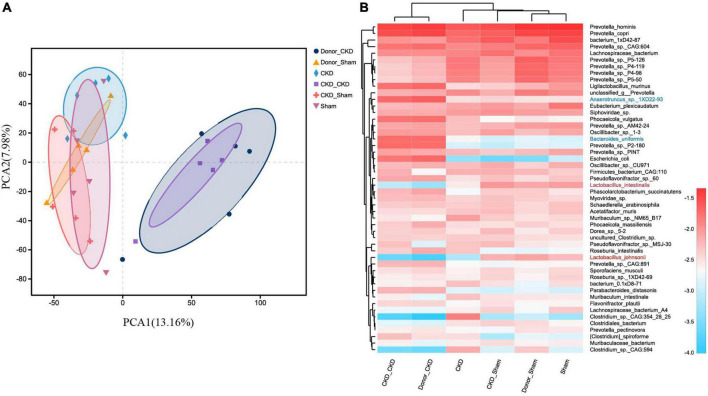
MGS changes after successful FMT. **(A)** PCA plots of species-level metagenomic expression (PERMANOVA *p* < 0.05). **(B)** Heat map of hierarchical clustering analysis of the differentially expressed species. The sample name and species name are shown on the lower and right side of the heat map, respectively, and the species clustering tree and sample clustering tree are shown on the left and upper side if cluster analysis was performed, respectively. The scale of the heat map means z-score.

**FIGURE 6:**
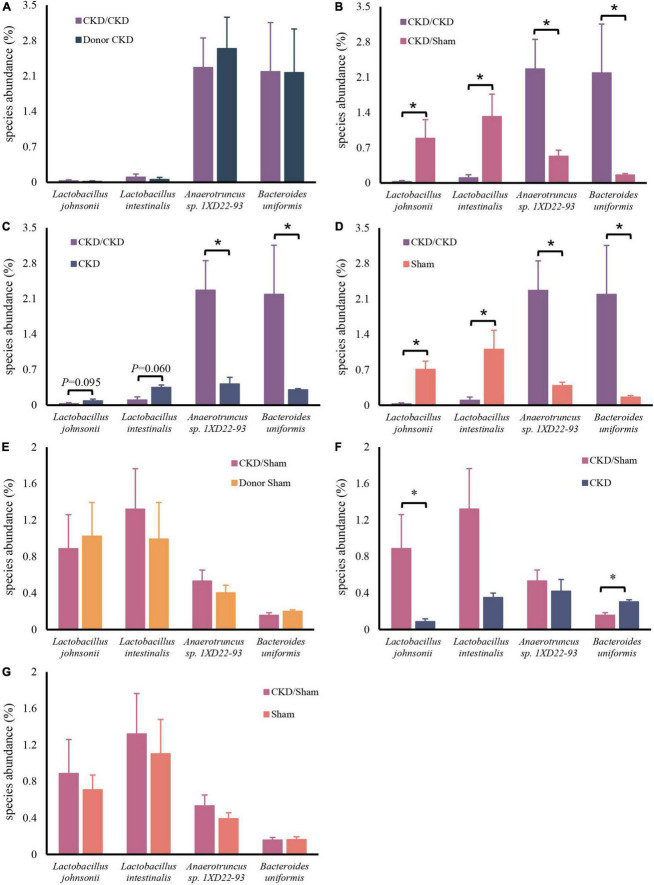
Differentially expressed species abundance among rats. **(A)** CKD/CKD VS Donor CKD. **(B)** CKD/CKD VS CKD/Sham. **(C)** CKD/CKD VS CKD. **(D)** CKD/CKD VS Sham. **(E)** CKD/Sham VS Donor Sham. **(F)** CKD/Sham VS Sham. **(G)** CKD/Sham VS CKD. The data is presented as means ± SEM. Donor Sham, *n* = 5; Donor CKD, *n* = 5; CKD/CKD, *n* = 5; CKD/Sham, *n* = 5; Sham, *n* = 5; CKD, *n* = 5. *P < 0.05.

After confirming the alteration of intestinal flora in rats with CKD, we examined whether FMT might restore the intestinal microbiota. The intestinal flora between recipients and donors reached homogeneity (PERMANOVA *P* < 0.05) after 1 week of FMT (Figure 5A). Furthermore, MGSs natural clustering were perfectly congruent with the experimental design grouping, indicating that the flora was significantly distinct across experimental groups and that donor flora were effectively colonized in recipients by FMT (Figure 5B). Meanwhile, the high abundance of *Lactobacillus johnsonii* and *Lactobacillus intestinalis* were colonized in the CKD/Sham group, but *Anaerotruncus* sp. *1XD22-93* and *Bacteroides uniformis* enriched in CKD/CKD group by heat map, suggesting that FMT successfully colonized the dominant bacteria in the organism (Figure 5B). Further analysis showed that the recipient has inherited, respectively, the characteristic bacteria from the donors, which differed significantly between two recipients (Figures 6A,B,E). By comparing the two recipient groups with the CKD group, the main flora differences were also reflected in the characteristic strains of the donors, respectively (Figures 6C,F). Moreover, compared to the Sham group, CKD/Sham could result in similar levels of strain abundance to Sham after FMT even though renal damage existed (Figure 6G). At the same time, comparisons of the CKD/CKD and Sham groups showed similar results to those of Donor CKD and Donor Sham (Figures 3C, 6D). In summary, FMT effectively modifies the original intestinal flora structure by donor gut microbiome.

### Fecal microbiota transplantation led to distinct amino acid metabolism

**FIGURE 7:**
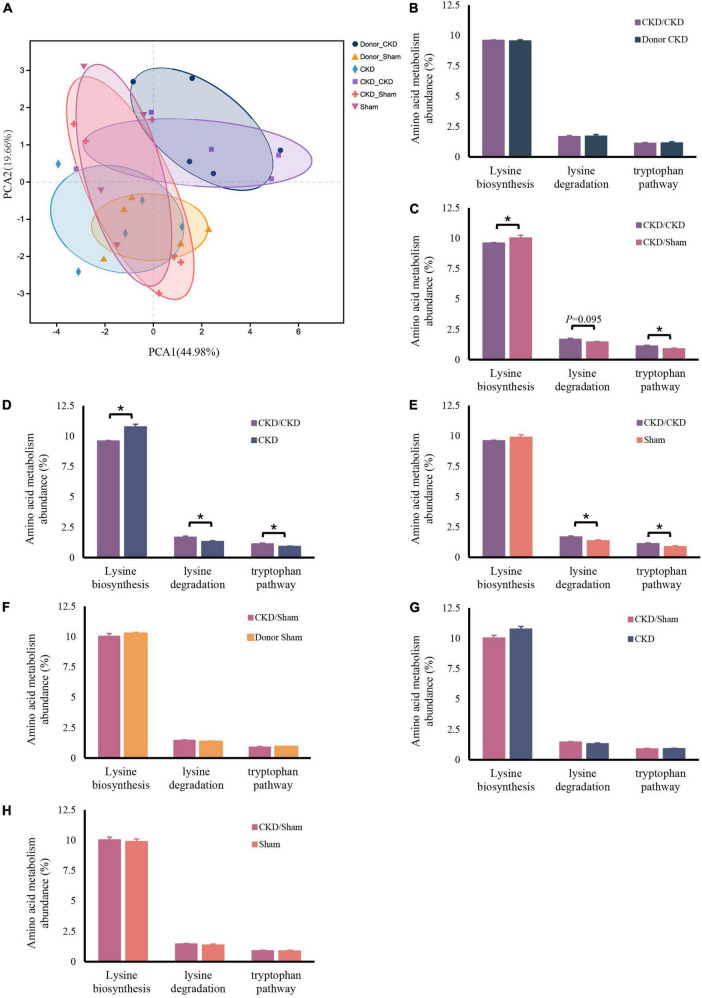
Changes in bacterial amino acid metabolism associated with the KEGG database after FMT. **(A)** PCA plots of amino acid metabolism expression (PERMANOVA *P* < 0.05). **(B–H)** Differentially expressed amino acid metabolism pathway abundance among rats. **(B)** CKD/CKD VS Donor CKD. **(C)** CKD/CKD VS CKD/Sham. **(D)** CKD/CKD VS CKD. **(E)** CKD/CKD VS Sham. **(F)** CKD/Sham VS Donor Sham. **(G)** CKD/Sham VS Sham. **(H)** CKD/Sham VS CKD. The data is presented as means ± SEM. Donor Sham, *n* = 5; Donor CKD, *n* = 5; CKD/CKD, *n* = 5; CKD/Sham, *n* = 5; Sham, *n* = 5; CKD, *n* = 5. *P < 0.05.

Similarly, the bacteria amino acid metabolism was strongly inherited (Figures 7A,B,F). The expressions of metabolic pathways involved in lysine breakdown and tryptophan metabolism were considerably elevated in CKD/CKD group (Figures 7C–E). Moreover, biosynthesis of lysine was active in all groups (Donor Sham, CKD/Sham, and Sham) of rats with healthy colonies and remained in same levels (Figures 7E–H). The more surprising correlation was in the CKD group. In the final analysis, a comparison of findings indicated that the abundance of amino acid metabolism in CKD was comparable to the level in CKD/Sham not in CKD/CKD. Based on this data, it was evident that abnormalities of intestinal amino acid metabolism in mild CKD develop slowly, but dysbiosis bacteria can cause rapid disruption of intestinal amino acid metabolism in mild CKD. Conversely, maintaining a normal intestinal flora can inhibit the abnormalities of intestinal amino acid metabolism in CKD. Therefore, timely correction of intestinal flora disorders in CKD can effectively prevent disturbances of bacterial amino acid metabolism from exacerbating disease progression.

### Fecal microbiota transplantation reduced the accumulation of protein-bound uremic toxins in chronic kidney disease

**FIGURE 8:**
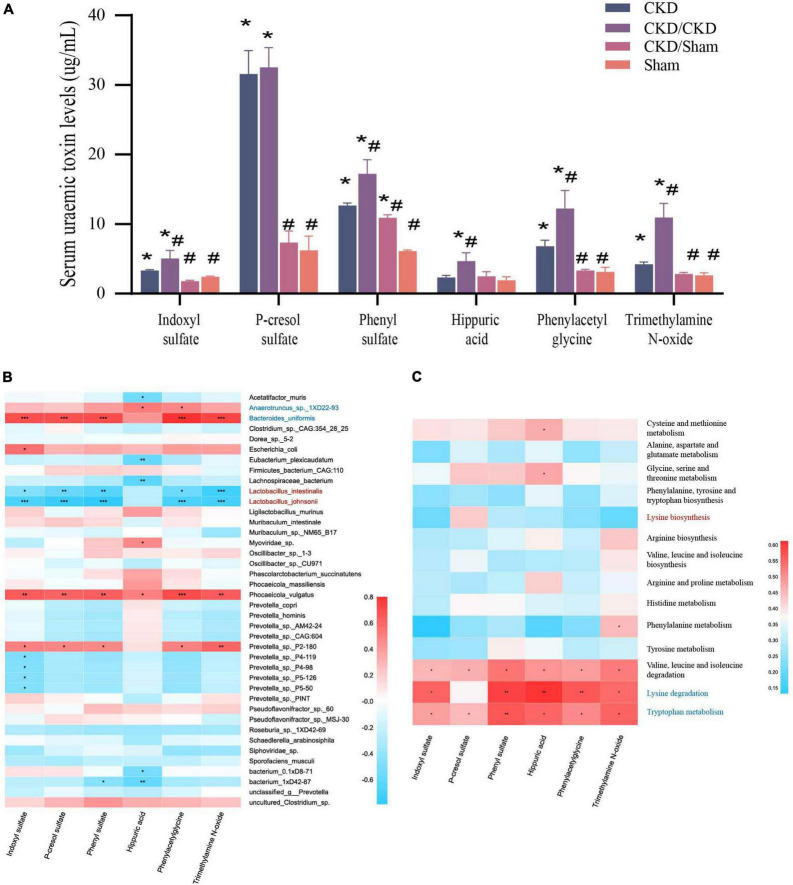
**(A)** PBUTs levels after FMT. The data is presented as means ± SEM. *n* = 5. *VS Sham group *P* < 0.05, ^#^VS group CKD *P* < 0.05. **(B)** Correlation heatmap between environmental factors and species with 1/2 nephrectomized after successful FMT. The X and Y axes are environmental factors and species, respectively, and the correlation coefficient *R*-values and the corresponding *P*-values were obtained by calculation. The *R*-values are shown in different colors in the figure, *P* < 0.05 is marked with *, and the right-hand legend shows the color intervals of different *R*-values. The clustering of species and environmental factors, respectively, is shown on the left and upper side of the figure. **(C)** Correlation heatmap between amino acid metabolism function and PBUTs with rats after successful FMT. The X and Y axes are environmental factors and amino acid metabolism function, respectively, and the correlation coefficient *R*-values and the corresponding *P*-values were obtained by calculation. The *R*-values are shown in different colors in the figure, *P* < 0.05 is marked with *, and the right-hand legend shows the color intervals of different *R*-values. The clustering of amino acid metabolism function and environmental factors, respectively, is shown on the left and upper side of the figure.

PBUTs in each group were at different level in the end of study (Figure 8A). PBUTs levels were substantially linked to flora in all groups following FMT (*r* = 0.31014, *P* = 0.004) (Table 2). The correlation heat map revealed that the effective colonization of main different bacteria (such as *Lactobacillus johnsonii* and *Lactobacillus intestinalis*) in healthy rats was significantly negative correlated with PBUTs (*P* < 0.05) (Figure 8B). In contrast, the dominant bacterium in CKD, *Bacteroides uniformis*, was strongly associated with increased PBUTs (*P* < 0.05) (Figure 8B). Thus, PBUTs in healthy colony receivers remained at the same level as in Sham, but the PBUTs in diseased flora recipients were substantially higher than others (*P* < 0.05) (Figure 8A). At the end of the experiment, the PBUT content of rats in the CKD group was significantly different from the other groups, but the numerical values were more nearly to CKD/Sham or Sham than to the CKD/CKD group. Such a trend was identical to the trend in amino acid metabolism in the intestinal flora of each group of rats at 1 week of FMT. Meanwhile, correlation heatmap between colonic flora amino acid metabolism and PBUTs once again validated the contribution of tryptophan metabolism and lysine degradation to the accumulation of PBUTs (Figure 8C). In summary, disrupted intestinal flora and aberrant bacterial amino acid metabolism enhance the accumulation of PBUTs in CKD, whereas FMT can correct the disturbance of the intestinal microenvironment to reduce the accumulation of PBUTs.

**TABLE 2:** Correlation of mantel test between gut microbiota and toxins after FMT.

dm1	dm2	Mantel r	*P*-value	Permutations	Tail type
Species matrix	Toxin matrix	0.31014	0.004	999	Two-sided

### Kidney function was improved by transplanting normal flora

**FIGURE 9:**
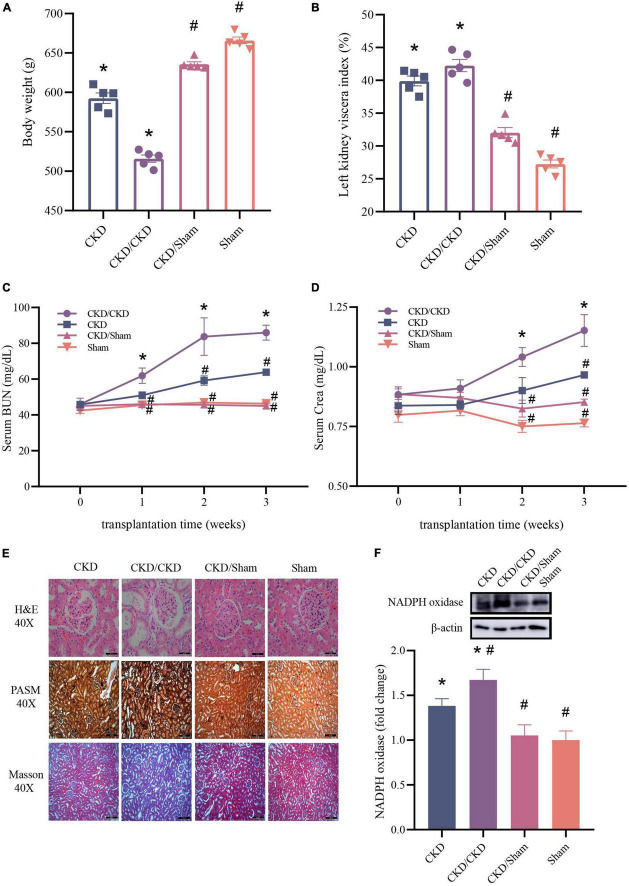
Changes of renal function related indicators after successful FMT. **(A)** Body weight. **(B)** Left kidney viscera index. **(C)** BUN. **(D)** Crea. **(E)** Histology of kidney tissue stained with H&E, PASM, and Masson. **(F)** Western blot results for NADPH oxidase. The data is presented as means ± SEM. *n* = 5. *VS Sham group *P* < 0.05, ^#^VS group CKD *P* < 0.05.

To evaluate the effect of FMT in CKD, the typical serum renal function indicators and histologic changes of kidney were measured. Results showed that rats received the malignant CKD colony exhibited considerable weight loss (*P* < 0.05), and the residual kidney exhibited hyperplasia (*P* < 0.05) (Figures 9A,B). One week after FMT, BUN and Crea levels of CKD/CKD group were close to those at the experimental end point in CKD group, and their renal function continued to decline over time (*P* < 0.05) (Figures 9C,D). Interestingly, in the same half-nephrectomized rats received the normal flora, kidney function did not appear to deteriorate and remained consistently at a similar level to the Sham group (Figures 9A–D). In agreement with the physiological parameters, CKD/Sham exhibited almost no histological damage of kidney. There was no vacuolar degeneration or apoptosis in renal cells and no pronounced glomerular shrinkage or cystic dilatation. Also, CKD-induced renal interstitial fibrosis was notably attenuated (Figure 9E). Moreover, significant decrease in NADPH oxidase protein level was observed in both CKD/Sham and Sham groups compared to CKD or CKD/CKD groups indicating that there was no sign of oxidative damage in CKD/Sham group (Figure 9F). In general, CKD related physiological indicators of rats exposed to healthy colonies showed no deterioration with time and remained at normal level.

## Discussion

It is becoming interesting that the interaction between host and its gut microbiota has important effect on the health of host (Valdes et al., 2018). Different microbiota in colon produces different PBUTs (Aronov et al., 2011; Wong et al., 2014; Fogelman, 2015; Tanaka et al., 2015), which are harmful to organs especially kidney (Bammens et al., 2006; Itoh et al., 2012; Ramezani et al., 2016). In the current study, we found that FMT from normal flora could improve gut microbial dysbiosis and change metabolite profiles, which could alleviate kidney damage and slow the progression of CKD.

Disturbance of intestinal flora existed in CKD and long-term flora disturbance will promote the deterioration of CKD (Mahmoodpoor et al., 2017). The harmful bacteria keep accumulating in colon as the progress of CKD (Al Khodor and Shatat, 2017). Therefore, severe CKD donor was simulated by 5/6 nephrectomy to explore accurate CKD representative bacteria. And through analyzing metagenomics between Donor CKD and CKD/CKD, it was found that *Anaerotruncus* sp. *1XD22-93* and *Bacteroides uniformis* may be involved in CKD bacterial dysbiosis. *Bacteroides uniformis* has previously been found to generate beta-lactamase (Tajima et al., 1983). And in earlier research findings, beta-lactamase could induce nephritis (Yousefichaijan et al., 2018). Meanwhile, in a separate investigation with the similar results, in patients with CKD, the enrichment of PBUTs increased as the number of Bacteroides uniformis increased, however, the underlying mechanism was unknown (Shivani et al., 2022). These findings all suggest that *Bacteroides uniformis* play a more critical role in CKD. At the same time, *Lactobacillus johnsonii* and *Lactobacillus intestinalis* were shown to be the most important contributing species in healthy colon compared to rats with renal disease. In a recent study, *Lactobacillus johnsonii* was shown to be able to reduce inflammatory response in human (Marcial et al., 2017). Results suggested that *Lactobacillus johnsonii* inhibited the microbial metabolism of tryptophan, preventing tryptophan from being transformed into harmful PBUTs and allowing it to be absorbed directly by the body. It indicated that in CKD, maintaining the homeostasis of *Lactobacillus johnsonii* may reduce the microbial metabolism of tryptophan, hence decreasing its conversion to toxic metabolites. Thus, *Lactobacillus johnsonii* deficiency may exacerbate the progress of CKD. Importantly, deficiencies of these beneficial species in the intestinal flora of CKD rats can be reversed by FMT from normal flora. Taken together, enrichment of harmful bacteria and loss of beneficial bacteria are the main flora characteristics of CKD, which are committed to the deterioration of CKD. Nevertheless, FMT from normal flora to renal disease can effectively colonize beneficial bacteria to reduce organism damage.

Pathogenic metabolome changes caused by alteration in the microbiome have been given a lot of attention in research on renal health (Chen et al., 2019; Wang et al., 2020; Wu et al., 2020). It is advantageous to understand their links in order to decrease the course of CKD. In a normal environment for gut flora, the gut flora metabolism creates few precursors of PBUTs (e.g., indole, p-cresol, and phenol). Nevertheless, the gut flora disrupted by CKD increases a large number of PBUTs precursors. These compounds are absorbed by gut and hasten the buildup of PBUTs in blood (Wang et al., 2020). According to previous research, these precursors are mostly produced from aromatic amino acids (Wikoff et al., 2009). To confirm that FMT modifies the whole gut microenvironment amino acid metabolic due to affecting the intestinal flora, we examined the amino acid metabolic pathway of KEGG in both the donor and recipient flora of FMT. Our results indicated that FMT of CKD/Sham group could effectively suppress the previously observed tryptophan metabolism related with the generation of PBUTs. Moreover, it was firstly discovered that lysine biosynthesis was highly active in normal gut flora, but lysine degradation was predominant in CKD. And in CKD/Sham group, FMT could reduce lysine degradation and boost lysine biosynthesis. Lysine is not involved in the synthesis of toxins, but is diuretic and promotes the elimination of toxins (Rubin et al., 1960). In addition, studies have shown that lysine administration provides renal protection in salt-sensitive hypertension (Rolleman et al., 2008; Rinschen et al., 2022). Thus, the significantly increased abundance of lysine degradation in the gut flora also contributes to worsening subsequent CKD. In our experiments, FMT from normal intestinal feces effectively replicated the enhanced synthesis of lysine in the donor flora and inhibited lysine degradation, thereby delaying the progression of CKD. Our data provide new ideas that inhibiting lysine degradation may reduce PBUTs, as opposed to the only reduction in tryptophan metabolism for PBUTs suppression. At the early stage of CKD, reducing PBUTs from their source can prevent the progression of CKD. In conclusion, FMT from normal feces enables beneficial bacteria to colonize in colon and the amino acid metabolic microenvironment is improved, leading to less metabolic load on kidney.

Several studies have been dedicated to reducing PBUT levels in CKD. However, only some kinds of toxins in PBUTs could be reduced and it’s hard to get back to normal level (Furuse et al., 2014; Guida et al., 2014; Ali et al., 2016; Rossi et al., 2016). Excitingly, our results showed that FMT was effective in maintaining blood main PBUTs at normal levels in CKD. Meanwhile, in the trial conducted by Barba et al. (2020) therapy of adenine-induced CKD with FMT from normal bacteria resulted in an improvement in PBUTs but did not prevent renal damage. This outcome is due to the fact that the effective development of a CKD model using adenine is a very violent procedure. When the model is effectively formed, the kidney is already seriously injured, and future deterioration is restricted (Diwan et al., 2018). In order to prevent such an occurrence, we have made a surgical mold by 1/2 nephrectomy, which ensures the lack of kidney function but also is easy to observe whether the remaining kidney transforms from a healthy to a sick one. By the end of the first week of our 3-week FMT, colonization of the flora was confirmed. At this time, in the CKD/Sham group, neither BUN nor Crea was elevated, and both of them always remained same levels over the ensuing 2-week course of the illness. In addition, kidneys showed no histological abnormalities at the end of the trial. In contrast, in CKD and CKD/CKD groups, the kidneys are in a state of continuous deterioration. Taken together, the therapeutic impact of FMT on CKD was not temporary but long-lasting. In the absence of significant organic lesions in CKD, FMT from normal flora prevents renal degeneration by reducing the accumulation of PBUTs in the blood. This suggests that FMT has the potential to be used clinically for long-term relief of CKD.

The findings of this experiment imply that FMT can be used to prevent CKD patients from acquiring malignant lesions, particularly without organic disease. Recent research has resulted in the establishment of uniform rules, processes for standardized donor selection, and procedures for standardized microbiological screening for stool preparation (Giles et al., 2019). The application of FMT is also becoming a more viable option. However, the limitation of this study was that the mechanism of protection was not explored further. This will also be a new entry point for future research on the treatment of CKD.

## Conclusion

In conclusion, the findings of this study indicate that restoring the intestinal environment with FMT is an effective therapy for CKD. FMT lowers the accumulation of PBUTs in the host by rectifying the gut microbiota amino acid metabolism, hence reducing the progression of CKD. Our findings also imply that inhibition of lysine degradation could be a therapeutic target for the reduction of PBUTs. In general, our data provide crucial evidence for the FMT treatment of CKD patients.

## Data availability statement

The original contributions presented in this study are publicly available. This data can be found at: NCBI repository, BioProject accession number: PRJNA874518.

## Ethics statement

The animal study was reviewed and approved by the Animal Experimentation Ethics Committee of the China Agricultural University.

## Author contributions

XL, XFW, XYW, and FR: conceptualization. XL, MZ, and YL: methodology. XL, PL, and LW: investigation. XL: formal analyses and writing—original draft. XL and MZ: visualization. MZ: validation. XFW: data curation. XL, MZ, XYW, and FR: writing—review and editing. XYW: project administration. FR: supervision. All authors have read and agreed to the published version of the manuscript.

## Footnotes

1
http://cloud.majorbio.com
